# Cardiopulmonary Resuscitation: Clinical Updates and Perspectives

**DOI:** 10.3390/jcm13092717

**Published:** 2024-05-06

**Authors:** Stephan Marsch, Timur Sellmann

**Affiliations:** 1 Department of Intensive Care, University Hospital, 4031 Basel, Switzerland; stephan.marsch@usb.ch; 2 Department of Anaesthesiology and Intensive Care Medicine, Bethesda Hospital, 47053 Duisburg, Germany; 3 Department of Anaesthesiology 1, Witten/Herdecke University, 58455 Witten, Germany

Cardiopulmonary resuscitation (CPR) stands as a cornerstone in emergency care, representing the crucial link between life and death for victims of cardiac arrest. Over the years, advancements in medical knowledge, technology, and training methodologies have propelled CPR into a dynamic field with continuous updates. In this closing editorial of the Special Issue “Cardiopulmonary Resuscitation: Clinical Updates and Perspectives”, we sum up the major findings of the published articles. Nineteen manuscripts were submitted for consideration for the Special Issue, with thirteen papers finally accepted for publication and inclusion (eleven research articles and two reviews). The contributions as an overview of the published articles are listed below. 

## 1. Simulation/Education

Effective CPR is an arduous task that requires skill, precision, and quick decision making—it is a stressful task per se. In view of the substantial evidence and the importance of simulation research, documented by high-ranking publications [1], innovative “high volume/high fidelity” simulation models are currently being developed. Models such as those enable us to measure the effectiveness of medical education and training methods in simulated CPR and to publish the results to increase the scope of available evidence and the visibility of scientific projects. Simulation models enable us to investigate relevant CPR questions using state-of-the-art methodology, avoiding methodological, logistical, and ethical challenges that would arise in studies with real patients. High-fidelity mannequins, virtual reality, and immersive scenarios nowadays enable healthcare providers to experience almost perfect realistic resuscitation situations. In this Special Issue, two papers on simulated cardiac circulation dealt with the effect of potential distractors on the overall performance of the teams during resuscitation. While both studies showed that randomly present relatives with different behavioral patterns (withdrawn or agitated) or carrying personal protective equipment (PPE) mentally stress the teams (measured by the NASA Task Load Index), no poorer performance during CPR could be demonstrated, at least for relatives’ presence. For PPE, on the other hand, both the stress level and the significantly poorer performance are surprising, as the data were generated in 2021, i.e., after more than 2 years of pandemic experience. This made it possible to present results that might not have been intuitively expected. Simulation remains an ideal tool for analyzing special situations (e.g., rare, extremely time-consuming, and time-sensitive scenarios). Schmitz and colleagues broke new ground with their study first describing resuscitation in microgravity. Scientific findings such as these have the potential to become part of current guideline recommendations, for example in the “CPR under special circumstances” chapter of the ERC [2], especially considering the increasing number of manned space flights. One logical further development would now be the integration of artificial intelligence to (a) either further improve the simulation scenarios, (b) further support teams during a simulated CPR, or (c) both. This individualized approach enhances competence and confidence in performing CPR, ultimately translating into improved patient outcomes.

## 2. Prehospital Care

The initial phase of cardiac arrest is most decisive for patients’ outcomes. As such, innovations in prehospital care are essential for improving overall survival rates. Different mobile technologies such as the integration of automated external defibrillators (AEDs) [3] into public spaces and the utilization of drone technology for rapid AED delivery to remote locations are transforming the landscape of prehospital care [4]. Additionally, telemedicine has enabled real-time communication between emergency medical services (EMSs) and healthcare providers, allowing for the early identification of potential cardiac events and the initiation of care even before the patient reaches the hospital [5]. In this context, four publications dealt with the prehospital treatment of cardiac arrest victims. Out-of-hospital cardiac arrest (OHCA) has a high prevalence of obstructive coronary artery disease and total coronary occlusion. Macherey-Meyer and colleagues were able to demonstrate for the first time that prehospital loading with antiplatelets and anticoagulants did not increase bleeding rates and was associated with favorable survival. They also found overtreatment of non-ischemic victims as well as undertreatment of ischemic victims, concluding that loading without definite diagnosis of sustained ischemia remains debatable until more data are available. Several skills have been identified as critical for ensuring patient safety such as communication and teamwork. The Team Emergency Assessment Measure (TEAM) questionnaire is designed to rate the non-technical performance of emergency medical teams during emergencies, e.g., resuscitation or trauma management. Originally developed in Australia, it has today been translated into and validated in more than 10 languages [6].

Han et al. were able to show high reliability (Cronbach’s alpha of 0.939; mean interitem correlation of 0.584) and a good concurrent validity (0.682), both indications of significant association (mean item–total correlation of 0.789), as well as excellent agreement (mean intra-class correlation coefficient of 0.804), making the TEAM approach a valid and reliable tool to evaluate the non-technical skills of a team of paramedics performing CPR. Regarding the selection of the target hospital, the research group of Jung and colleagues once again succeeded in highlighting the importance of the cardiac arrest centers (CACs) recommended in the current guidelines. Thus, patients in the CAC group had a significantly higher likelihood of good neurological recovery and survival to discharge compared to the non-CAC group. The fact that only around a quarter of the approximate 96,000 patients were transported to CACs is somewhat saddening. We remain hopeful that the results of this work will help to change this. Finally, Chiang et al. offered more insight into the ongoing debate about the role of mechanical CPR devices in OHCA, concluding that their use may benefit adults who have suffered OHCA in achieving ROSC and survival to admission. But they also concluded that due to the low certainty of evidence, more well-designed large-scale randomized controlled trials are needed to validate these findings.

## 3. Post-Resuscitation Care

Post-resuscitation care plays a crucial role in neurological outcome and overall recovery. As the incidence of OHCA is high and the survival rate is low, it remains a challenge to treat those who survive the first phase and regain spontaneous circulation. In a previous narrative review, the authors summarized the existing evidence on oxygen dosage, the role of noradrenaline in urine output and blood pressure, and blood pressure targets themselves. Furthermore, they offered the prospect of experimental approaches to improve neuroprognostication, which includes various approaches like bundles and novel biomarkers. While whole-blood transcriptome analysis has been shown to reliably predict neurological survival in two feasibility studies, Hinkelbein et al. were unable to prognosticate survival or non-survival after OHCA based on proteomics-based serum alterations in the human protein expression, at least not in the first 24 h after ROSC. However, since a good neurological outcome is essential, advancements in neuroprognostication as well as the integration of neuroimaging, biochemical markers, and electrophysiological assessments are vital to refine prognostic accuracy and thus aid clinicians in making informed decisions regarding the continuation or withdrawal of aggressive interventions [7]. Injuries related to CPR are a frequent finding in cardiac arrest victims. These include bony and parenchymal organ injuries [8]. The most common injuries frequently found are chest wall injuries [8], but if and to what extent these CPR-associated chest wall injuries contribute to a delay in the respiratory recovery of cardiac arrest survivors has not been sufficiently explored. Kunz and colleagues examined surviving intensive care unit (ICU) patients who had undergone CPR due to medical reasons in their single-center retrospective cohort study in relation to the existence of CPR-associated chest wall injuries, detected by chest radiography and computed tomography. Over a third of all included patients presented with chest wall injuries, including flail chest in 9%. Through the multivariable logistic regression analysis, the authors were able to identify flail chest to be independently associated with the need for tracheostomy (OR 15.5). In the linear regression analysis, pneumonia and fractured ribs were associated with an increased ICU length of stay, whereas flail chest and pneumonia were associated with a prolonged duration of mechanical ventilation. It was interesting to see that four patients with flail chest who underwent surgical rib stabilization could be weaned successfully from the ventilator afterwards. This means that surgical treatment of rib fractures could represent a valuable therapeutic option in this population. As the results of this study suggest, CPR-associated chest wall injuries, flail chest in particular, may impair the respiratory recovery of cardiac arrest survivors in the ICU. These findings make this study a valuable addition to the body of clinical data but need to be confirmed in larger trials. 

## 4. Excursion: Controlled Automated Reperfusion of the Whole Body (CARL)

**Figure 1:**
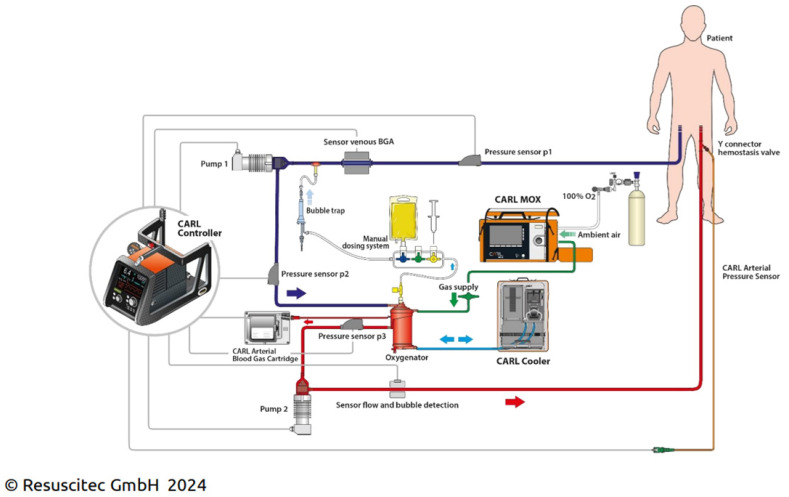
A schematic representation of the CARL (Controlled Automated Reperfusion of the Whole Body) operating principle. The right to use the image was granted by Prof. Dr. med. Georg Trummer as a consultant of Resuscitec GmbH for the “Medical” sector. BGA: blood gas, MOX: mobile oxygenator.

In the context mentioned above, the CARL system offers interesting and physiologically comprehensible and fascinating approaches, which have also been positively confirmed in initial reports. CARL therapy (or targeted extracorporeal CPR) consists of core elements such as the control of physical reperfusion conditions (blood pressure, blood flow, pulsatility, and blood temperature), the situational modification of the reperfusion solution, usually recirculating the blood, by adjusting the oxygen and carbon dioxide content, the pH value, the electrolyte content, and the osmolarity, as well as quickly available, comprehensive monitoring. The focus is on preparing the tissue damaged by the lack of oxygen for the resumption of the body’s own blood circulation [9] (see also Figure 1). The CARL concept constitutes a new frontier in resuscitation science by focusing on the controlled restoration of blood flow following cardiac arrest, where a pulsatile flow is used instead of a continuous flow within the framework of an ECPR, aiming to minimize reperfusion injury and optimize organ recovery within an individualized therapeutic regimen. This approach involves the precise regulation of blood pressure, oxygenation, and perfusion rates during the reperfusion phase [9]. CARL represents a paradigm shift, challenging traditional beliefs about the immediate and aggressive restoration of blood flow. By carefully managing the reperfusion process, CARL seeks to mitigate the harmful effects associated with the abrupt reintroduction of oxygen to ischemic tissues. Ongoing research and clinical trials are exploring the potential of CARL to enhance survival rates and improve long-term outcomes in post-cardiac arrest patients. In this Special Issue, the research group led by Trummer published two experimental studies. The first study compared the CARL model directly to ECPR in refractory OHCA. By using CARL, not only were spontaneous rhythm conversions observed using a modified priming solution, but the application of potassium-induced secondary cardioplegia also proved to be a safe and effective method for sustained rhythm conversion. Finally, significantly fewer defibrillation attempts were needed, and cardiac arrhythmias were reduced during reperfusion via CARL. In their second animal model study, the authors were able to demonstrate a highly significant favorable neurological outcome for animals with head elevation during the first 20 min of post-resuscitation care, whereas histopathologic findings did not show corresponding differences between the groups. A possible explanation for the transient neurologic deficits potentially attributable to functions localized in the posterior perfusion area may be venous congestion and edema as modifiable contributing factors of neurologic injury following prolonged cardiac arrest. We hope that the results of this interesting technique in humans are just as promising as those obtained so far in the animal model.

## 5. Pediatrics

While the principles of CPR remain consistent across age groups, pediatric resuscitation presents unique challenges and considerations. Recognizing the differences in anatomy, physiology, and the underlying causes of cardiac arrest in children is paramount for successful outcomes [10,11]. Educational programs must address the specific needs of healthcare providers dealing with pediatric populations, emphasizing the importance of age-appropriate techniques and interventions [10,11,12]. Birth asphyxia is a major cause of delivery room resuscitation. Subsequent organ failure and hypoxic–ischemic encephalopathy (HIE) account for 25% of all early post-natal deaths. In neonatal sepsis, the nSOFA (neonatal sequential organ failure assessment) considers platelet count as well as respiratory and cardiovascular dysfunction. In this Special Issue, Dathe et al. evaluated the nSOFA for the first time in cases of resuscitation regarding its potential as a useful predictor for in-hospital mortality in neonates (≥36 + 0 weeks of gestation) following asphyxia with HIE and therapeutic hypothermia. The nSOfA scores for survivors were lower overall, as were their respiratory, cardiovascular, and hematologic sub-scores compared to non-survivors. The odds ratio for mortality was 1.6 [95% CI = 1.2–2.1] per one-point increase in the nSOFA and the optimal cut-off value of the nSOFA to predict mortality was 3.5 (sensitivity of 100.0%; specificity of 83.9%). With the help of their results, the authors were able to prove that an early nSOFA (≤6 h of life) offers the possibility of identifying infants at risk of mortality. This is an important finding, since early accurate prognosis following asphyxia with HIE and therapeutic hypothermia is essential to guide decision making.

## 6. Conclusions

Cardiopulmonary resuscitation remains a dynamic field, constantly evolving to incorporate the latest clinical updates and perspectives. Simulation and education, post-resuscitation care, prehospital interventions, pediatric considerations, and emerging concepts like Controlled Automated Reperfusion of the Whole Body collectively contribute to the advancement of CPR practices. As we navigate this ever-changing landscape, collaboration between healthcare professionals, researchers, educators, and technology developers remains crucial. By staying informed and embracing innovation, the medical community can continue to refine and optimize CPR protocols, ultimately improving outcomes for individuals facing cardiac arrest. This Special Issue highlights many aspects of many areas of resuscitation (from preclinical to post-resuscitation) over a wide age range (from neonates to adults) in many different types of studies (from retrospective to prospective studies, and from original research to review papers and meta-analyses). In conclusion, the diverse array of topics covered in this Special Issue underscores the multifaceted nature of cardiopulmonary resuscitation, emphasizing the ongoing pursuit of excellence in saving lives across various age groups and clinical scenarios. We extend our gratitude to all contributors for their invaluable insights, and we look forward to the ongoing collaboration and innovation that will further advance the field of resuscitation medicine.
